# VEP Correlates of Feedback in Human Cortex

**DOI:** 10.1371/journal.pone.0051791

**Published:** 2012-12-12

**Authors:** Yury Petrov, Jeffrey Nador, Jiehui Qian

**Affiliations:** Psychology Department, Northeastern University, Boston, Massachussets, United States of America; Dalhousie University, Canada

## Abstract

It is known that neural responses become less dependent on the stimulus size and location along the visual pathway. This study aimed to use this property to find evidence of neural feedback in visually evoked potentials (VEP). High-density VEPs evoked by a contrast reversing checkerboard were collected from 15 normal observers using a 128-channel EEG system. Surface Laplacian method was used to calculate skull-scalp currents corresponding to the measured scalp potentials. This allowed us to identify several distinct foci of skull-scalp currents and to analyse their individual time-courses. Response nonlinearity as a function of the stimulus size increased markedly from the occipital to temporal loci. Similarly, the nonlinearity of reactivations (late evoked response peaks) over the occipital, lateral-occipital, and frontal scalp regions increased with the peak latency. Response laterality (contralateral vs. ipsilateral) was analysed in lateral-occipital and temporal loci. Early lateral-occipital responses were strongly contralateral but the response laterality decreased and then disappeared for later peaks. Responses in temporal loci did not differ significantly between contralateral and ipsilateral stimulation. Overall, the results suggest that feedback from higher-tier visual areas, e.g., those in temporal cortices, may significantly contribute to reactivations in early visual areas.

## Introduction

The event-related potentials technique (ERP) has been actively used in brain research for the last half-century. The technique relies on interpreting peaks and troughs in the time course of electric potentials measured on the scalp (EEG). Because the ERP features usually reflect integrated activity of multiple cortical sites interpretation of their neurophysiological bases is problematic making the ERP technique very empirical [1]–[4]. Although source localization allows to infer cortical sources of ERP with some degree of certainty [5], it does not explain the functional meaning of the ERP features. Ideally, one would like to interpret the features not only as neural activity in a given brain area, but also to understand its functional origin: whether it is an intrinsic activity or it results from interactions with other areas, e.g., feedforward or feedback interactions. Causality analysis can in principle be applied to such problems [6]–[8], but because ERPs normally constitute only a fraction of the on-going EEG activity it is likely that causality measures will be dominated by causal interactions within the endogenous EEG activity (brain rhythms, etc.) rather than by the exogenous event-related potentials. In this study we propose a different approach to this problem based on analyzing the amount of response saturation with stimulus area within the ERP waveform.

**Figure 1:**
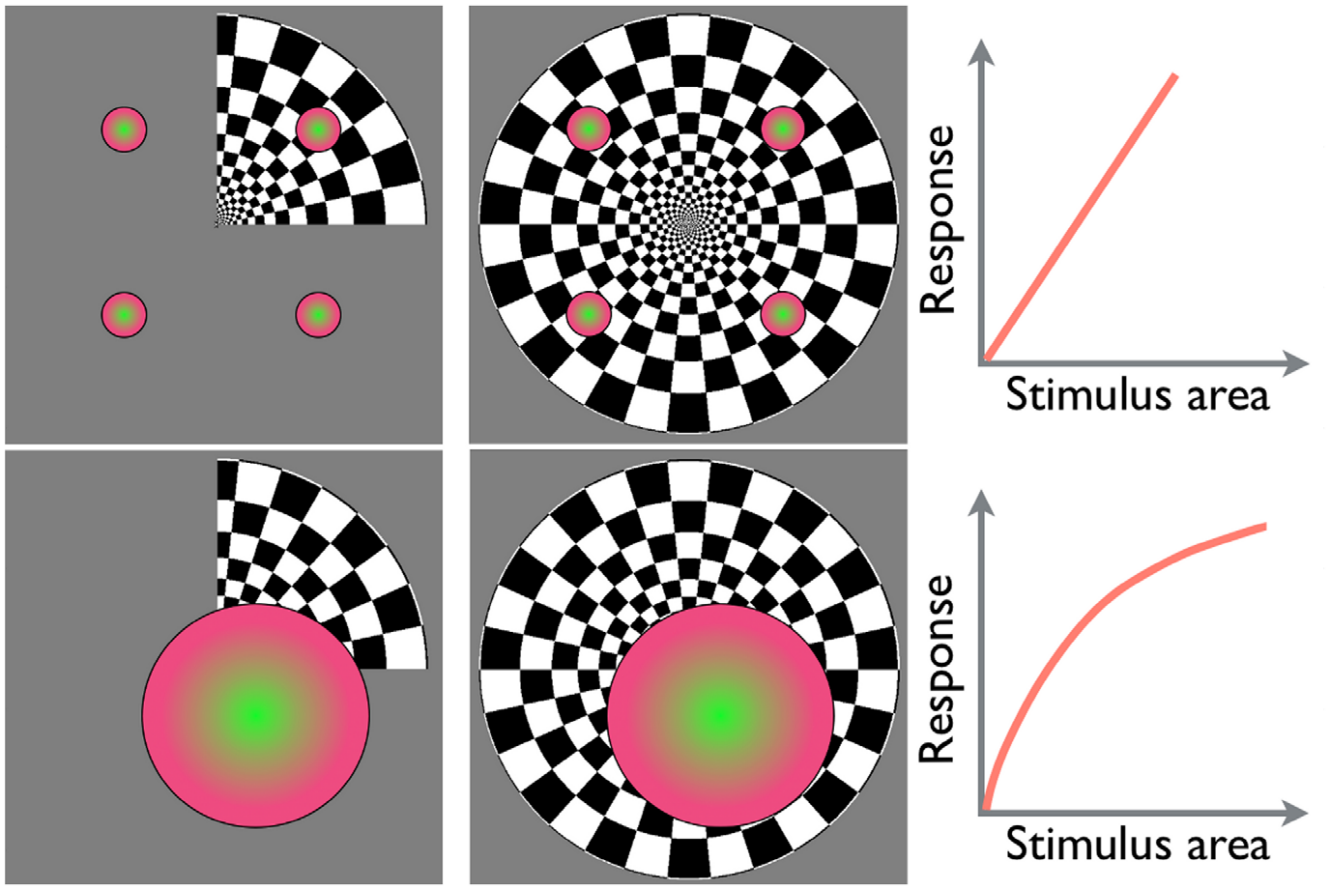
Top: small receptive fields. A larger stimulus engages more neurons; VEPs for individual quadrants sum up linearly to the full stimulus VEP. Bottom: large receptive fields. A larger stimulus engages the same neurons; neuronal responses saturate, VEPs for individual quadrants sum up nonlinearly to the full stimulus VEP. The neuronal response input–output nonlinearity is illustrated on the right.

One potential reason for the response saturation is that receptive field sizes increase along the visual processing stream. Classical models of the visual system’s organization have characterized its function primarily in terms of feedforward processing [9]. According to such models, neurons at each hierarchical stage of processing collect their inputs from neurons at a previous stage and pass their outputs to neurons at the next stage. Typically, the higher-level neurons integrate the outputs of multiple lower-level neurons with nearby or overlapping receptive fields. Consequently, receptive field sizes are expected to increase at later stages of visual processing. This was confirmed for retinotopic areas by primate single cell studies [10]–[17] and human fMRI experiments [18], [19]. When receptive fields of neurons are much smaller than the stimulus size, a larger stimulus engages a proportionally larger population of neurons. Their combined outputs simply add-up as the stimulus size increases. Consequently, VEPs evoked by a full-field stimulus should equal the sum of VEPs evoked by four one-quadrant stimuli presented one by one. This point is illustrated by the top panel in Figure 1. This is true notwithstanding neuronal input–output nonlinearity and in spite of VEP source cancellation, which happens when local cortical currents flow in opposite directions. The latter point follows from the principle of superposition for linear conductors, which applies to head tissues [20]: electric potentials produced by multiple current sources simply add up, i.e., their lead-field is linear. Hence, the cancellation of scalp potentials due to mutually facing cortical sources happens exactly the same way regardless of whether the sources were active simultaneously or in separate trials, as long as the EEGs for these separate trials were summed up.

On the other hand, when receptive fields are comparable or larger in size than the stimulus, the additional stimulus area amounts to a stronger input to the same population of such neurons (e.g., neurons in higher-tier areas). This makes the sigmoid input–output nonlinearity typical for neurons (Figure 1, bottom panel) appear in the resulting VEP. Hence, VEPs evoked by a full-field stimulus will be weaker than the sum of VEPs evoked by four one-quadrant stimuli for these higher-tier areas. This is unlike the VEP signal from the underlying lower-tier units with small receptive fields.

Along with the receptive field sizes other factors contribute to the response saturation. Evoked responses due to higher-order visual mechanisms, such as boundary processing or object recognition are not necessarily proportional to the object’s size. In the case of periodic stimulation higher brain areas can show a predictive activation which might happen even before the stimulus appears. This predictive response might have little to do with the stimulus size or intensity. Instead, it might reflect cognitive processing associated with detection of trial onset; attention allocation, change detection, suppression of saccades, etc.

In this study we varied the stimulus size and measured the degree of the VEP response saturation for several scalp ROIs. The measure was then used as an indicator of higher-tier visual areas to analyse VEP waveforms over early visual areas in terms of feedback from the higher-tier areas. Hence, the precise nature of the VEP response saturation was not important for this study.

Response laterality can serve as another indicator of feedback. It is well documented that receptive fields of neurons in inferior temporal cortices of monkeys are not only very large but also usually extend into both visual half-fields [14]–[17]. Conversely, receptive fields of neurons in early visual areas are small and rarely extend into the ipsilateral visual half-field [11]–[13], [21]. In this study we compared VEP responses between contralateral and ipsilateral stimulation for temporal and lateral-occipital scalp loci and interpreted the relative degree of ipsilateral response as an indicator of feedback from higher-tier visual areas, possibly those in temporal cortices.

## Materials and Methods

### Ethics Statement

The study was approved by the Northeastern University Institutional Review Board and was conducted in accordance with its guidelines. A written informed consent (approved by the IRB) was obtained from all human subjects.

### Stimuli

**Figure 2:**
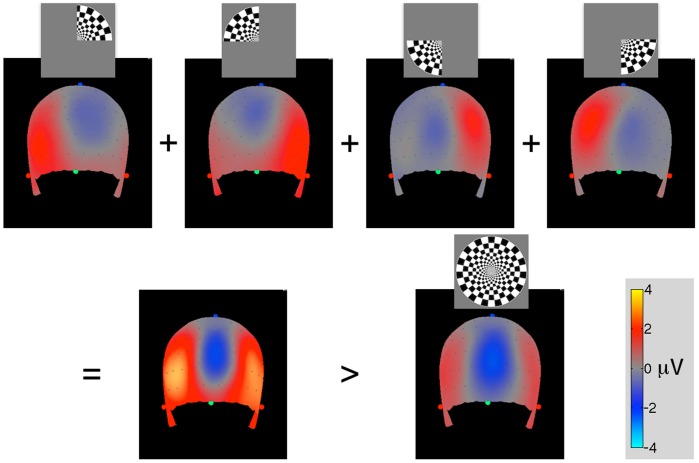
Subject-averaged scalp VEPs at 100 msec after stimulus onset. The back of the head is shown. Green and red dots indicate the nasion and tragi points respectively. Top: responses to individual quadrant stimuli. Bottom: sum of the four quadrant responses compared to the full-disk response.

**Figure 3:**
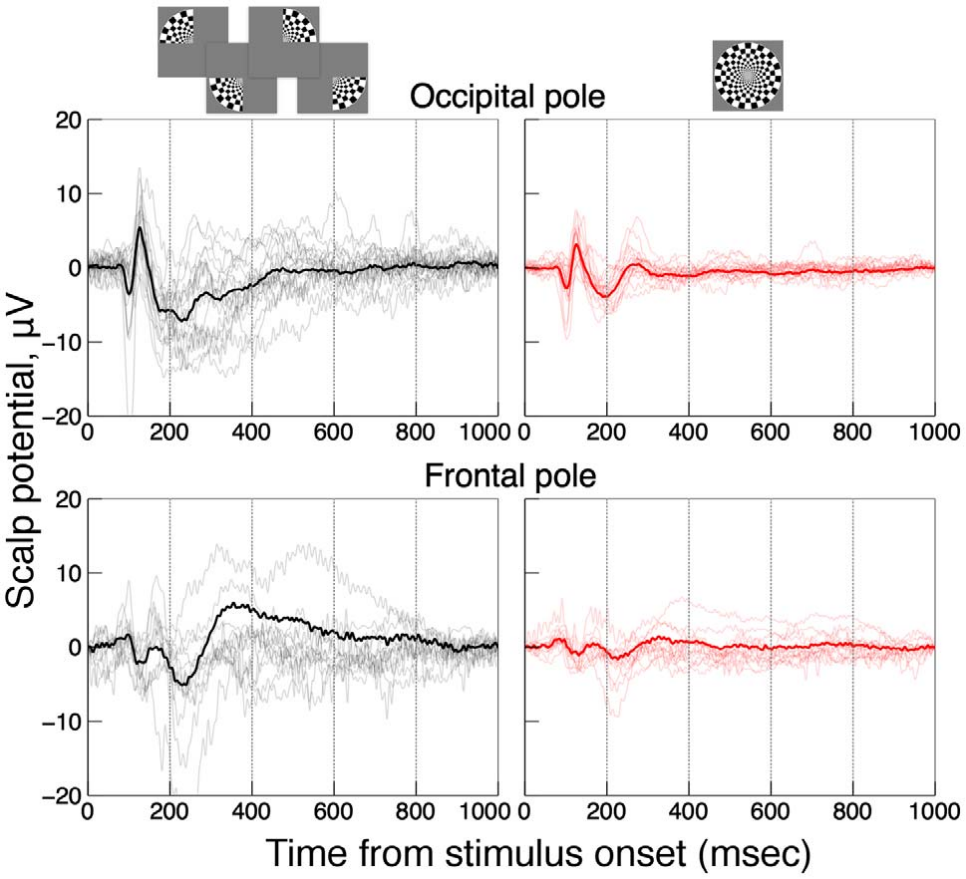
Scalp VEPs at an occipital electrode (top panel) and a frontal electrode (bottom panel). Individual subject’s VEPs are shown by thin traces, the averaged VEPs – by bold traces. Black curves show the sum of four VEPs, one for each quarter-disk stimulus, red curves show full-disk VEPs.

**Figure 4:**
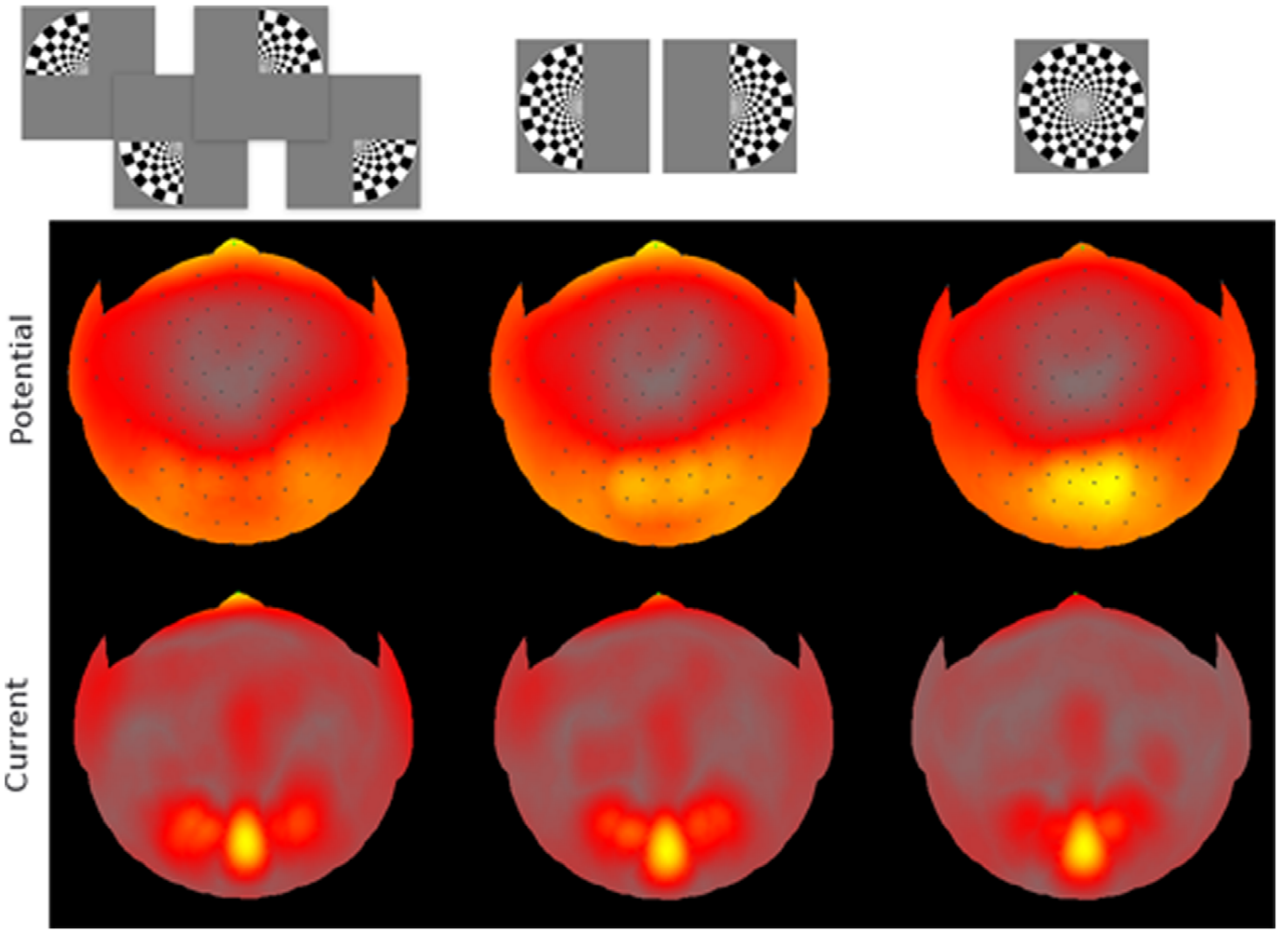
Maximal amplitudes of scalp VEPs (top) and skull-scalp currents (bottom) averaged across subjects. The maximum activity distributions are shown on flattened scalp surface. Dark dots on the scalp surface indicate electrode locations. The top (North) of each surface map corresponds to nasion, the bottom (South) – to the occipital pole area. Colormaps are not on the same scale across the panels, instead each panel’s scale was set to its full range of amplitude variation. The ranges were (for panels from left to right) 9.8, 5.9, 3.7 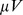 for scalp potentials, and 10.9, 7.8, 6.1 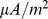 for skull-scalp current densities.

The stimulus was a 100% contrast checkerboard grating, 16° in diameter displayed on a medium gray background equated in luminance to the checkerboard stimulus. Seven stimulus configurations were used, they differed only in the area of the visual field stimulated: a full-disk grating, a left half-disk grating, a right half-disk grating, and quarter-disk gratings occupying one of the 4 quadrants of the visual field each. A small fixation cross was shown at the center of the screen at all times. The stimuli are illustrated by the insets in Figures 2, 3, and 4.

After the onset of the checkerboard stimulus it reversed contrast every second for 10 seconds followed by a 3 second rest interval. Only the fixation mark was displayed on the gray background during the rest interval. The seven stimulus configurations were presented in random order, 200 stimulation epochs (1000 msec each) were displayed for each stimulus configuration; the entire experiment lasted for approximately 30 minutes.

The stimuli were generated using an in-house visual psychophysics library (PEACH). They were viewed on a linearized 21″ ViewSonic G225f monitor. The monitor resolution was set to 1600×1200 pixels; for the used viewing distance of 70 cm, a pixel subtended 1 minute of arc. The monitor refresh rate was 75 Hz.

### Data Acquisition

EEG was recorded using HydroCell GSN 128-channel nets, amplifiers, and the accompanying NetStation software by EGI Inc. Electrode locations on the head and head landmarks (nasion and tragi points) were measured for each subject using a Polhemus FASTRACK digitizer and in-house software (3Digit). EEG epoch markers were recorded by the NetStation software synchronously with the EEG data acquisition using a DIN signal generated by PEACH via the DATAPixx peripheral (VPixx Technologies). The markers were generated at the beginning of the first CRT raster sweep after the stimulus onset or contrast reversal, which were taken as the beginning of an ERP epoch in our study. The stimulus rendering was completed 13.3 msec later at the end of a video frame. EEG data acquisition was externally triggered by the DATAPixx peripheral synchronously with the monitor refresh signal (75 Hz), the trigger rate was set to 7 samples per videoframe resulting in 525 Hz sampling frequency. EEG and event markers data were saved on a hard drive and processed off-line.

### Data Processing and Analysis

Data processing and analysis were performed using an in-house MATLAB software suite (Harmony). EEG potentials were average referenced over all channels. AC line noise frequencies (60 Hz and 120 Hz) were notch-filtered out. Constant and linear terms were subtracted from raw epochs to remove DC components and occasional amplifier reset artifacts. 5% to 25% of raw epochs were rejected due to muscle activity artifacts determined by potential thresholding. Because subjects were encouraged to blink during the rest intervals the rejected epochs often included the first epoch after each rest interval. The remaining epochs (approximately 180 per condition) were averaged for each subject. Noisy electrodes were identified based on the proportion of the rejected epochs, and, if detected, were replaced by thin-spline interpolating data from the remaining electrodes [20, Appendix J]. The experimental noise variance on each electrode and its covariance among electrodes (the noise covariance matrix) were estimated by projecting out the averaged epoch from each raw epoch and then averaging the remaining signal covariance over the epochs.

Across-subjects data averaging was performed using scalp interpolation. Electrode locations on each scalp were measured with respect to the subject’s head landmarks (nasion and tragi points, see Figure 2). The electrode positions were then averaged across subjects to find averaged electrode locations. Each subject’s EEG data were then interpolated from the actual electrode locations to the averaged electrode locations using the thin-plate 3D spline interpolation. Finally, the resulting interpolated data were averaged across subjects.

To improve spatial resolution of our analysis we used a surface Laplacian method (also known as deblurring or scalp current density, SCD). This technique aims to reduce strong blurring of EEG potentials resulting from abrupt electric conductivity changes at the boundaries of the skull and the surrounding tissues [20], [22], [23]. Another advantage of this method is that it avoids ambiguities of potential referencing because unlike electric potential electric current is reference-free. The surface Laplacian method transforms scalp potentials to the corresponding electric currents at the skull-scalp interface. Conventional source localization methods cannot uniquely determine brain sources of EEG from the recorded scalp signals and therefore depend on *a priori* constraints (such as minimum-norm, smoothness, number of sources, etc.). Conversely, the surface Laplacian provides a unique solution for the skull-scalp currents best matching the observed EEG distribution on the scalp in a parameter-free fashion except for an overall scaling factor [23]. For this reason the method is also sometimes called “downward continuation”.

Importantly, no anatomical MRI images were necessary for the transformation to the skull-scalp currents. The only used parameter was the scaling factor casting the surface Laplacian of the scalp potential into the units of electric current density on the skull-scalp interface. This factor depends only on the head radius, scalp thickness, and scalp conductivity [23]. The scaling factor was calculated based on the average head radius of 9.2 cm, scalp thickness 0.5 cm, and its conductivity 0.3 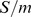. The values were based on the available anatomical data [20, chs. 4 and 6] and also in [24]–[27]. The skull-scalp currents were calculated by first fitting the measured scalp potentials with spherical harmonics [28] and then “downward continuing” the fit onto the skull-scalp interface using the analytical solution for each spherical harmonic [23]. The resulting deblurring of scalp VEPs is illustrated in Figure 4.

### Subjects

Twenty observers (11 females, 9 males) 24–40 years of age, with normal or corrected visual acuity participated in the study. Three of the observers were the authors. The remaining observers were naive to the purpose of the study and did not take part in EEG experiments before. Observers were trained for a short time to get acquainted with the stimuli and the task. They were instructed to fixate at the fixation cross at the center of the screen at all times and to minimize head and eye muscle activity during the visual stimulation intervals (10 seconds long). Otherwise, no task was performed. Five subjects showed excessive eye-muscle activity (blinks and eye movements) and their data were excluded from the study.

## Results

In the first part of this section responses to the quadrant and half-disk stimuli located in the left and right visual half-fields were pooled so that response nonlinearity as a function of the stimulus area was analyzed irrespectively of the stimulus location. Conversely, in the second part responses to the quadrant and half-disk stimuli were pooled so that response laterality (contralateral vs. ipsilateral) was analyzed irrespectively of the stimulus area.

### Response Nonlinearity as a Function of the Stimulus Area

Scalp VEPs 100 msec after the stimulus onset averaged across observers are shown in Figure 2. Evoked potentials for individual quadrants are shown in the top row, their sum is shown in the bottom row where it can be compared with the VEPs evoked by the full-disk stimulus. Electrode potentials were interpolated over the averaged scalp surface using the thin-plate 3D spline method [20, Appendix J]. The occipital pole negativity represented by the cool colors changed only slightly between the sum of the quadrants and the full-disk stimuli unlike the occipito-temporal positivity represented by the hot colors, which was much weaker for the full-disk stimulus. This result indicated stronger response saturation for lateral activations compared to occipital activations.

Figure 3 shows averaged VEP epochs at a mid-occipital, 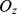, site (top row) and a mid-frontal-pole, 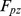, sites (bottom row). The epoch time is plotted along the x-axis, VEPs – along the y-axis. Time zero corresponds to the stimulus onset, i.e., to the start of the contrast reversed stimulus rendering onto the screen. Individual subjects’ data were plotted by thin traces, the averaged data were plotted by bold traces. The sum of quadrants VEPs were plotted on the left (in black), the full-disk VEPs were plotted on the right (in red). There were pronounced differences between the two conditions indicating response saturation at the frontal pole location: the full-disk stimulus produced a much weaker response than the sum of the quadrants stimuli. For the mid-occipital site, on the other hand, the differences are small during the first negative ERP peak (at 100 msec), but become more pronounced at greater latencies.

The maximal absolute values over the VEP epoch at each location are shown in Figure 4. The maximum maps are useful for finding hotspots of activity. The scalp potentials are shown in the top row, the skull-scalp currents – in the bottom row. Maximum maps for the sum of quadrants, sum of half-disks, and full-disks are arranged in columns. Hotter colors indicate larger amplitudes. Note that the colormaps are not on the same scale across the panels, instead each panel’s scale was set to its full range of amplitude variation. This allows to better compare the activity patterns across the three stimulus sizes. One can see that as the size increased from the quadrant to full-size, the activity weight shifted from frontal and temporal scalp areas to the occipital areas indicating stronger response saturation in the frontal and temporal areas.

**Figure 5:**
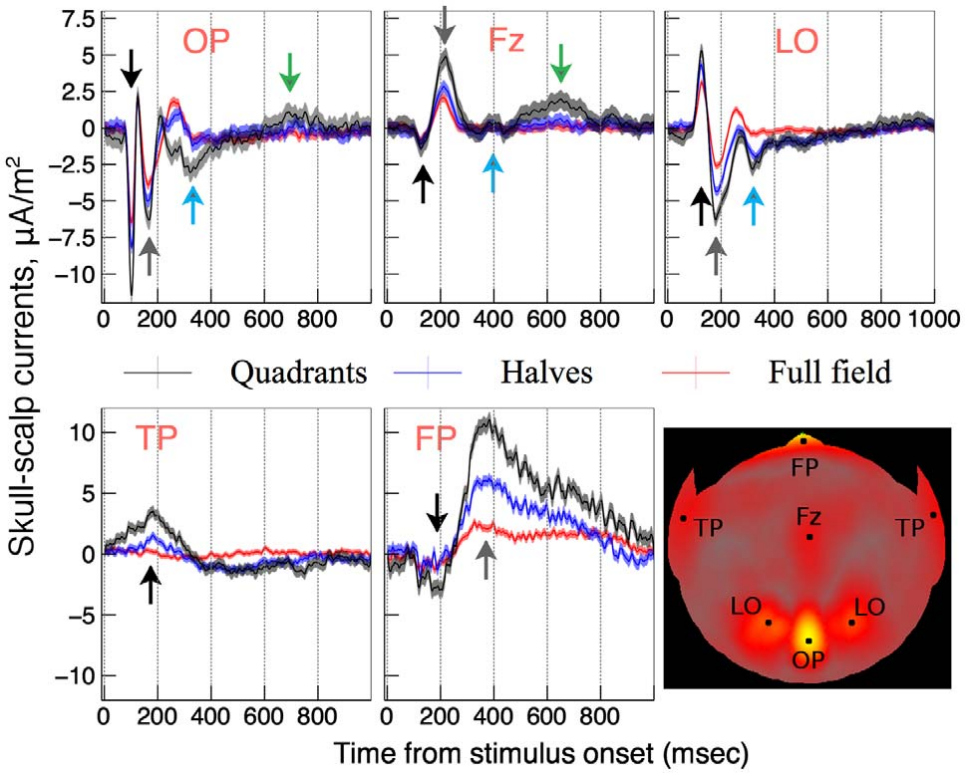
Averaged response epochs at the activation hotspots (see the inset). Quadrant, half-disk, and full-disk VEPs are shown in black, blue, and red respectively. Error bars showing the standard error of the mean (SEM) represent average response variation across the raw epochs. Peaks chosen for further analysis are indicated by the black arrow (the earliest peak) and gray, blue, and green arrows (later peaks).

Skull-scalp current patterns (the bottom row of Figure 4) were more focused than the corresponding VEP patterns, and several distinct activation hotspots could be identified. The foci were chosen based on the maximal skull-scalp currents in all seven conditions (4 quarter-disks, 2 half-disks, and 1 full disk). The following hotspots were chosen as regions of interest (ROIs, see the inset in Figure 5) based on the largest response magnitudes: the occipital pole (OP), the two adjacent locations to the left and to the right (LO), the location anterior to the center (Fz), two locations over the temporal pole areas (TP), and the frontal pole (FP). Note that the last two hotspots appear elongated in Figure 4 because of the flattening projection used here. OP, Fz, LO, and FP hotspots were readily apparent in all 7 conditions, while the TP hotspots were apparent in all but the full disk condition (Figure 4, bottom row). Because we did not use source localization and instead used skull-scalp current ROIs, the corresponding cortical ROIs could only be inferred. For instance, we speculate that the Fz hotspot reflects the frontal eye fields activity, while the LO hotspot could reflect any combination of MT, V4, or LOC activity.

Because responses to quadrants and half-disk stimuli located in left and right half-fields were pooled for the purpose of the present analysis there were no significant differences between signals for the left and right LO locations, and the corresponding data were averaged. Similarly, the data were averaged for the left and right TP locations. Figure 5 shows time course of the skull-scalp currents at the five scalp ROIs. The ROIs are marked by the black dots on the flattened scalp surface shown in the figure’s inset. Skull-scalp currents for the sum of quadrants, the sum of half-disks, and the full-disk stimuli are shown in black, blue, and red respectively. The following trends can be observed: (i) differences among the three traces increased from the occipital locations (OP, LO) to the temporal (TP) and frontal (FP) locations. Fz data appeared similar to these for OP and LO; (ii) the differences increased over time at all locations.

**Figure 6:**
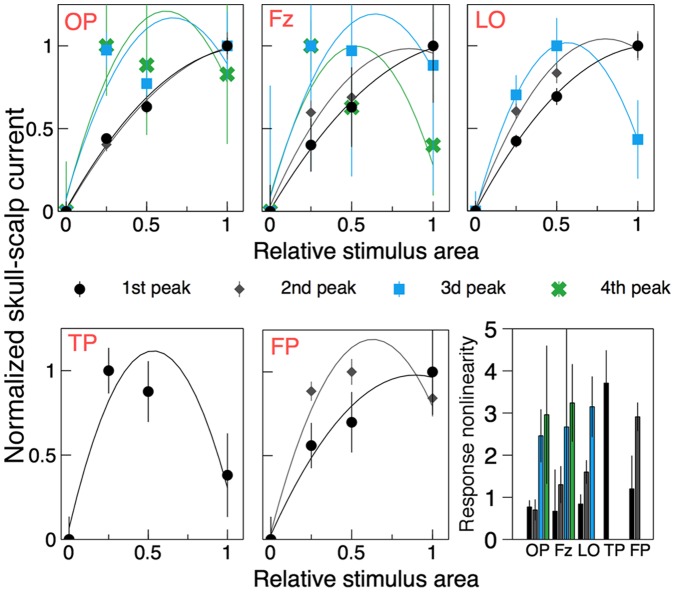
Amplitudes of the skull-scalp current peaks (normalized values) as a function of the relative stimulus area. Data for peaks of different latencies are shown with different colors, the corresponding peaks are shown in Figure 5 by arrows of the same color. Solid curves show quadratic polynomial fits to the data. The response nonlinearity measure, 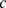, is plotted in the bottom right panel for all peaks.

To quantify (i) the magnitude (absolute value) of the first clearly identifiable peak was plotted vs. the stimulus area in Figure 6 (black disks). The peak was indicated by black arrows in Figure 5. The peak’s magnitude was averaged over the four quadrants for the quadrant stimulus and averaged over the two half-disks for the half-disk stimulus. Zero area (no stimulus) response was assumed to be zero for all ROIs. Data in Figure 6 were normalized by the maximum value for each dataset and fitted with a quadratic polynomial 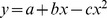. The fits are displayed with solid curves. The quadratic polynomial was chosen because it fitted the data well and provided a straightforward measure of the response nonlinearity in the form of the fitted coefficient 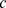. 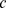 values were plotted in the last panel of Figure 6. The error bars show one standard deviation as estimated from the linear least-squares fitting. The response nonlinearity of the first peak shown with black bars was relatively weak for OP, Fz, and LO indicating little saturation. For FP the nonlinearity was larger but not significantly so. For TP, on the other hand, the response was significantly more nonlinear than for other ROIs (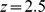, 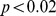). Moreover, the TP response not only saturated but actually decreased as the stimulus area increased.

To quantify (ii) we repeated the above analysis for other peaks, wherever they could be clearly identified for at least two stimulus sizes. These peaks were indicated by gray, blue, and green arrows in Figure 5. Curves of the same colors were used to plot the respective polynomial fits in Figure 6. The response nonlinearity 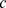 increased significantly for later peaks in all ROIs. As was the case for TP, other peak magnitudes saturated and then decreased as the stimulus size increased for the longest latency peaks in all ROIs.

**Figure 7:**
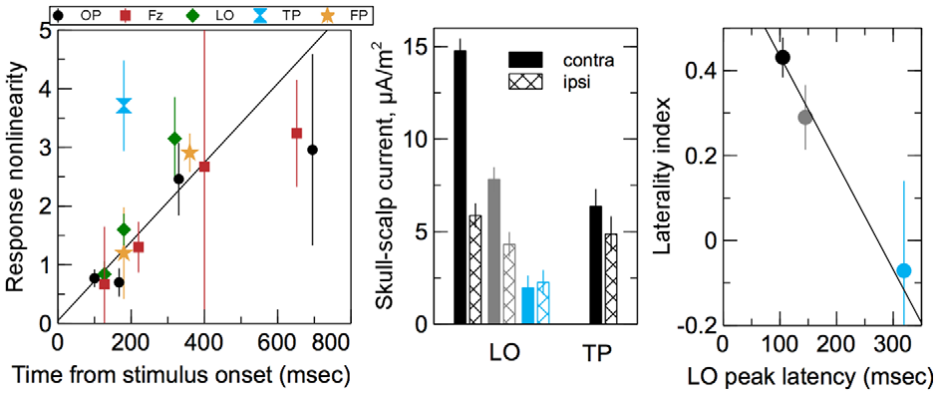
Left: Response nonlinearity shown in the last panel in **Figure 6** plotted as a function of the time from the stimulus onset for the corresponding peaks. The solid line shows a linear fit to the data compounded over all ROIs except TP. Middle: amplitudes of the three LO peaks and the TP peak compared between contralateral (solid bars) and ipsilateral (hashed bars) stimulation. The bars are color coded by the same colors as the respective peaks in Figure 5. Right: the laterality index for the three LO peaks plotted as a function of the peak latency. The solid line shows a linear fit to the data.

The fitted 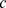 values were plotted as a function of the respective peak latencies in the left panel of Figure 7. The TP datum shown with the blue symbol was a clear outlier characterized by the strong early nonlinearity. The remaining data fell onto a single trend line indicating an increase of the response nonlinearity with peak latencies. To measure the significance of the trend all data excluding the TP datum were compounded and fitted with a linear polynomial fit shown with the dashed line. The intercept of the fit was not significantly different from zero (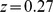, 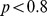), its slope, on the other hand, was highly significant (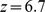, 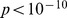).

### Response Laterality in LO and TP Regions

The left and right LO and TP ROIs were well separated on the scalp and hence skull-scalp currents evoked in response to contralateral and ipsilateral stimulation could be compared for these loci. First, responses to the individual quadrant and half-disk stimuli in the respective (contralateral or ipsilateral) visual half-fields were summed. Then, the results were averaged between the left and right ROIs. The resulting amplitudes for the first three LO peaks indicated in Figure 5 by black, gray, and blue arrows and for the TP peak indicated by the black arrow were plotted in the middle panel of Figure 7. The bars were color coded using the same scheme as for the respective peaks in Figure 5. The contralateral and ipsilateral responses were not significantly different in TP. In LO, on the other hand, the contralateral response was much stronger for the first peak, somewhat stronger for the second peak, and about the same as the ipsilateral response for the third peak.

To quantify the observed differences between contralateral and ipsilateral responses we defined the laterality index, 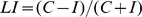, where 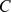 and 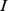 stand for contralateral and ipsilateral peak amplitudes respectively. 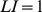 indicates a purely contralateral response, 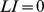 indicates a perfectly bilateral response. The laterality index for the three LO peaks was plotted as a function of the peak latency in the right panel of Figure 7. Datapoints were color coded using the same scheme as for the respective peaks in Figure 5. The solid line shows a linear fit. The slope of the fit was significantly different from zero (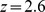, 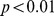), which establishes the statistical significance of the observed shift from the mostly contralateral response for the earliest peak to the mostly bilateral response for the last peak in LO.

## Discussion

In this study the effect of the stimulus size and location on VEP magnitude was measured at different scalp ROIs. Early VEP peaks showed little response saturation at occipital locations, but the saturation increased via lateral scalp regions toward temporal and frontal regions. This trend can be seen in Figures 2, 3, and 4. The maximal maps of VEPs (top row in Figure 4) demonstrate the saturation as a shift of relative activation from temporal and frontal scalp regions to occipital regions as the stimulus area increases. This trend could be also observed for skull-scalp currents (bottom row in Figure 4), which were calculated from the scalp potentials by the ‘downward continuation’ technique. The method produced a marked deblurring of the signals and allowed to identify well-defined hotspots of activation shown in the Figure 5 inset. The skull-scalp currents at the hotspots had distinct time courses both in terms of the peaks timings and their relative amplitudes, even when the ROIs were juxtaposed. For example, the LO currents were not merely opposite-direction versions of the OP currents, which would have been the case if they were side-lobes of the OP activations (Figure 5). This demonstrates that signals from nearby cortical areas can be recovered from strongly correlated scalp potentials by the deblurring procedure used.

Amplitudes of the first clearly identifiable peaks at the chosen ROIs were analysed to measure the response nonlinearity (saturation) across various scalp regions. The nonlinearity increased significantly from weak saturation for the regions above early visual areas (OP, Fz, LO) to strong saturation over temporal poles (Figure 6). The TP response was, in fact, not merely saturating but decreasing with the stimulus size to the effect that there was hardly any peak left for the full-disk stimulus (Figure 5). We can only speculate on the possible causes of such behavior. The weak response to the full-disk stimulus could result from a very strong long-range surround suppression or some other contextual effect in TP. Quadrants and, to a lesser degree, half-disk stimuli have L-junctions in their boundaries, which the full-disk stimulus is lacking. This could make the latter less “interesting” for the visual processing happening in TP. Also, it is lacking any texture boundary in central vision, which could also weaken the TP response.

The reactivation (late) peaks indicated by gray, blue, and green arrows in Figure 5 were used to measure response nonlinearity as a function of time from the stimulus onset. For the earliest reactivation peak (gray arrow) a tendency for response saturations was observed for all scalp ROIs except OP. This tendency increased dramatically for later reactivation peaks in the OP, LO, and Fz regions. The corresponding response curves plotted in blue and green in Figure 6 show that the responses were largely independent of the stimulus size (excluding the no-stimulus condition). Moreover, the latest reactivation peaks in all areas decreased with the stimulus size to the point where they were nearly absent for the full-disk stimulus. A measure of the response nonlinearity was calculated and plotted as a function of the peak latency in Figure 7. TP, where the response nonlinearity was atypically high for the observed peak latency, was a clear outlier. Given that TP also was the only ROI where the characteristic decreasing response was observed for the very first peak, it appears that TP holds a special place among the studied ROIs. For the rest of the ROIs the response nonlinearity showed a highly significant tendency to increase with the peak latency. Interestingly, the trend line was common for all the ROIs.

There are several possible explanations for the increase in the response saturation with the peak latency. VEP response can saturate with time as a result of neuronal adaptation through the course of the VEP epoch. We find this unlikely for two reasons. First, the stimulation was repeated every second for 30 minutes, and one would expect all adaptation to occur in the first few minutes of the experiment or even prior to the experiment during the training session. Second, given the small receptive field sizes (confirmed by the weak response saturation in OP, for example) the adaptation magnitude would be expected to be the same for all three stimulus sizes and therefore would not produce the observed saturation effect.

Alternatively, surround suppression could produce the observed response saturation as the stimulus area increased. However, neurophysiological [29] and psychophysical [30] studies have shown that surround suppression is the strongest shortly after the stimulus onset and then quickly decreases within the first 200 msec. Because the reactivation peaks which showed strong response saturation had latencies longer than 200 msec, the surround suppression explanation would predict a decrease in response saturation – opposite to what was observed.

Instead, our results suggest that the reactivations of early visual areas observed as the later VEP peaks are due to a feedback from higher-tier visual areas such as cortical areas in temporal lobes. Because the TP peak precedes the reactivations peaks by 150 msec (LO), 180 msec (FP), and 470 msec or more (OP, Fz), we hypothesize that these peaks have their origin in a feedback from the TP area. This explains the strongly increased response nonlinearity for the later peaks. Three results in particular support this hypothesis: (i) due to its early nonlinear response TP is clearly different from the rest of the ROIs; (ii) the measure of response nonlinearity plotted in the last panel of Figure 6 was the same for the early TP peak and all the succeeding peaks in the remaining ROIs (3d and 4th peaks in OP an Fz, 3d peak in LO and FT); (iii) similarly, the characteristic response decrease with the stimulus size of the TP peak was later observed for the succeeding peaks in the rest of the ROIs.

This hypothesis is further supported by the laterality analysis of the LO and TP responses presented in Figure 7. While the earliest LO peak was primarily evoked by contralateral stimulation, the contralateral advantage decreased for the second peak and completely disappeared for the third peak. In this respect the third LO peak was similar to the TP peak, where no significant contralateral advantage was observed.

Numerous experimental findings indicating feedback interactions in the visual system of human and nonhuman primates provide support to the feedback hypothesis. Single-cell recordings in nonhuman primates have shown that inactivation of higher-order areas modulates neuronal responses in lower-order areas [31]–[34]. For example, [33] have found that V1 activity is modulated by GABA inhibition of area V2. In another study [35] found similar results for V1, V2, and V3 neurons when area MT was inactivated. Many studies indicate feedback signals mediating surround suppression of V1 neurons [29], [36]. Taken together these results strongly support the role of feedback from higher visual areas in determining V1 neural activity. Feedback interactions in human vision were also reported recently. [37] have found that early (40–100 ms) inactivation of V1, using TMS, inhibits detection of simple features, but not conjunctions. Conversely, inactivation of V1 after longer delays (200–240 ms) seems to impair detection of feature conjunctions, while leaving simple feature detection intact. This double-dissociation implicates V1 in feedback loops with higher visual areas, although it does not specify where such feedback might originate. Other TMS studies [38], [39] specifically indicated feedback inputs from MT to V1 with latencies 80–125 msec from the stimulus onset.
